# Implementation of Lean Management Tools Using an Example of Analysis of Prolonged Stays of Patients in a Multi-Specialist Hospital in Poland

**DOI:** 10.3390/ijerph20021067

**Published:** 2023-01-07

**Authors:** Agnieszka Zdęba-Mozoła, Remigiusz Kozłowski, Anna Rybarczyk-Szwajkowska, Tomasz Czapla, Michał Marczak

**Affiliations:** 1 Department of Management and Logistics in Healthcare, Medical University of Lodz, 90-131 Lodz, Poland; 2 Centre for Security Technologies in Logistics, Faculty of Management, University of Lodz, 90-237 Lodz, Poland; 3 Department of Management, Faculty of Management, University of Lodz, 90-237 Lodz, Poland

**Keywords:** Lean Management, lean healthcare, length of stay, waste, efficiency

## Abstract

Healthcare institutions in Poland constantly encounter challenges related both to the quality of provided services and to the pressures associated with treatment effectiveness and economic efficiency. The implemented solutions have a goal of improving the service quality of lowering the continuously increasing operational costs. The aim of this paper is to present the application of Lean Management (LM) tools in a Polish hospital, which allowed for the identification of prolonged stays as one of the main issues affecting the service costs and the deteriorating financial results of the hospital. The study was conducted in the neurology department and involved an analysis of data for the whole of 2019 and the first half of 2022. In addition, surveys were conducted among the medical staff to help identify the main causes of prolonged stays. Methods of data analysis and feasible solutions were developed in order to improve the economic efficiency of the unit. The analysis shows that the application of LM tools may contribute to improvement in the functioning of hospitals and that further studies should focus on the development of the method to evaluate efficiency of the implemented solutions intended at shortening the hospital stays of the patients.

## 1. Introduction

Due to the growing pressure on financial results (constantly increasing debts of public hospitals in Poland) [1] and the shortages of qualified medical personnel, improvements in patient flow organization have more and more often become an area of interest in healthcare units. This is a field where improvements may result in both cost reductions and in a significantly higher quality of care. Waste reduction, efficiency improvements and patient safety trigger many activities aimed to improve the patient flow [2]. The data show that a markedly shortened length of stay (LOS) influences the number of admissions and the patient turnover rate, which has a positive effect on a hospital’s management and costs [3]. The length of stay is a general measure of hospital efficiency, commonly associated with a cost reduction when the LOS is reduced [4]. In addition, the length of stay and the waiting time in healthcare institutions are two factors that may directly affect the assessment of patient satisfaction. In out-patient settings, prolonged waits have a negative impact on patient satisfaction. Improvements in the clinical efficiency and elimination of wasteful practices may shorten the waiting time.

To increase their efficiency, hospitals implement Lean Healthcare (LH) processes with an emphasis on waste elimination and on providing a higher value to patients. The term “lean” originated in the Toyota Production System (TPS), which aimed to improve process efficiency. The TPS entered the medical sector at the beginning of 2000 and is commonly known as “lean healthcare”. The application of the TPS was considered an effective strategy for improving results and reducing costs by increasing the effectiveness of clinical care in hospitals. A U.S. study has shown that 70% of hospitals implement LH or similar approaches [5]. LH classifies activities into non-value-added (NVA) or value-added (VA); the VA activities aim at meeting patients’ needs while the NVA activities are associated with unnecessary space, time or resources [6,7]. In addition, LH helps to identify the NVA activities and to take measures in order to reduce or eliminate them. Similarly, wastes represent anything which is not the minimum room, equipment, staff and time necessary to add value to a service or product. In the literature, a view is presented according to which LM can be applied in any process in a healthcare facility to provide measurable benefits, such as reduction in inventory, shorter duration of activities and processes, improved quality of services or a higher level of satisfaction among both patients and employees [8]. Regardless of the manner and place of Lean implementation, five steps of its implementation are immutable and have to be followed in order: (1) define value from the patient’s perspective, (2) map the value stream and identify issues and constraints, (3) remove the wastes and ensure the value flows without interruptions, (4) implement the solution and let the patients pull value, (5) maintain the improvements and seek perfection [9,10,11].

Lean Healthcare contributes to improvements in the patient flow and hospital care efficiency [5]. In a neurology department, LH may help to standardize the process of a stroke center, effectively utilize medical resources, improve medical quality and reduce the cost of a single treatment [12]. It should be emphasized that when patients are considered ready for discharge, a greater LOS is associated with increased morbidity, mortality, peripheral venous complications and nosocomial infections [13], which results in decreased revenue for the hospital and negatively impacts patients’ safety and experience [13]. Moreover, other related benefits such as cost reductions, better return on investments and greater savings are observed with the decreased LOS [5,14,15,16,17].

The aim of the paper is to present financial benefits resulting from the identification of the factors which influence prolonged hospital stays in a neurology department of one Polish hospital, to indicate the Lean Management tools that help to identify these factors and to implement the measures to reduce the length of patients’ stay in the hospital.

## 2. Materials and Methods

### 2.1. Overview of the Background of the Project

The study was a continuation of the project completed at the J. Gromkowski Provincial Specialist Hospital in Wroclaw in 2019 and presented in the article titled Implementation of Lean Management in a Multi-Specialist Hospital in Poland and the Analysis of Waste [18]. During the project, several wastes were identified, which most frequently included seeking and explaining, waiting and overproduction. The next stages of the project enabled the identification of key scopes and locations where next actions had to be taken in order to improve the process. One of them was the problem of prolonged patients’ stays in the hospital. As a patient’s stay depends on a series of both medical and organizational conditions, this issue was selected as the subject of the current study.

The process of conducting the study is presented in Table 1.

The study discussed in this paper began in February 2022 and was initiated by defining the term of a prolonged stay and by identifying the department where the proportion of prolonged stays was considerably higher compared to the overall number of hospital stays.

For the study’s purpose, literature was reviewed concerning the term of prolonged stay. According to the Central Statistical Office, the average length of a patient’s stay in neurology departments in Poland was 6.7 days in 2019 [19]. Moreover, classification of a prolonged stay in emergency departments as over 7-day hospitalization can be found in the literature [20]. Therefore, a prolonged hospitalization of a patient was assumed to be a stay in the department lasting over 7 days.

The Neurology Department with the Stroke Subunit where a total of 1795 patients were admitted in 2019 and the percentage of prolonged stays was 44% was selected for the study. The department specializes in the diagnosis and treatment of nervous system diseases. Patients are mostly admitted due to vascular diseases, nervous system infections, nervous system injuries and tumors, as well as neuropathies and epileptic disorders. Moreover, the department is involved in drug programs for patients with multiple sclerosis on the basis of one-day stays. The department provides 35 beds. In 2019, the mean monthly occupancy rate was 100%, and it was higher than for other departments of the hospital by 24% on average. During the analysis, one-day stays were excluded, as they were not directly related to hospitalization, but to a specific medical procedure performed in the department (e.g., the drug program in accordance with the requirements of the payer, i.e., the National Health Fund). These stays could considerably distort the study’s findings.

**Scheme 1:**
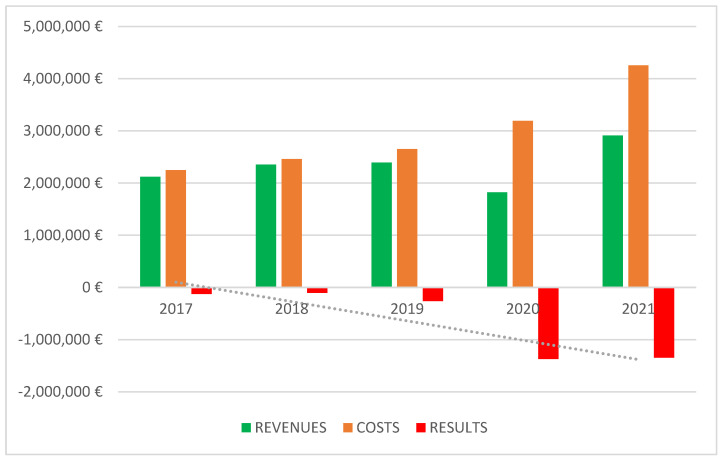
The financial results of Department III between 2017 and 2021.

In addition, financial data were checked for possible economic consequences of prolonged stays. In 2019, the overall department’s revenues were EUR 2,391,786.28 (PLN 11,264,835.00) and the costs were EUR 2,651,109.60 (PLN 12,486,196.00), which means a loss of EUR 259,323.33 (1,221.361,00 PLN) throughout the year. The financial results decreased compared to 2017 and 2018 and they additionally deteriorated in 2020 and 2021. The financial situation of the Department III in specific years is presented in Scheme 1.

For the study’s purpose, the hospital stays recorded in 2019 were analyzed. Starting from 2020 and the COVID-19 pandemic outbreak, the department was subjected to continuous transformations and reorganizations to increase the number of beds for patients with confirmed or suspected SARS-CoV-2 infection. Therefore, the data on lengths of stay in the department in 2020 and 2021 would contain distorted information.

### 2.2. Preparation of Medical Data

The stays related to drug programs were excluded from the overall cases of patients’ hospitalization in the department in 2019. These stays are related to one-day hospitalization to administer a medication in the treatment of a specific disease included in the special therapeutic program. Diagnoses with the highest percentage of cases requiring hospital stay (over 80%) when considering the overall stays in the department were selected for the study. The diagnoses included in the medical records that were analyzed for the study purpose are listed in Table 2.

### 2.3. ABC Analysis

**Scheme 2:**
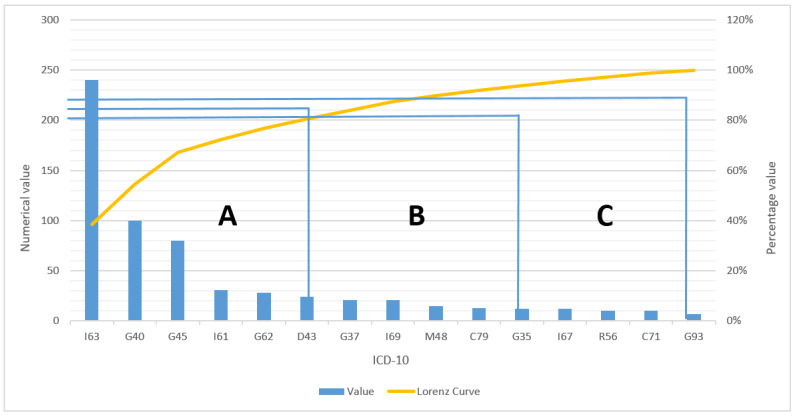
ABC analysis of the occurring diseases according to ICD-10.

Using the ABC method, the data and diagnoses were analyzed and the diagnoses for which the percentages of prolonged stays were the highest were classified into three groups. The group classification is presented in Scheme 2. The group A diagnoses were selected for further stages of the research.

### 2.4. Analysis of Medical Records

The next step was an analysis of medical records of specific patients with the group A diagnoses whose stays in the department were longer than 7 days. The records were anonymized and contained information on the patient’s sex and age, hospital admission type, main diagnosis, comorbidities, a history of medical procedures and rehospitalization cases, information about the patient’s death during hospitalization and the patient’s number in the hospital register book. The records did not contain any personal information to identify the patient. Based on the analysis, a form was developed where the following information was recorded:

- The date of discharge;
- The diagnosis;
- The procedure;
- The information on readmission;
- Whether the patient died or not;
- The patient’s age;
- The length of stay;
- The revenue;
- The cost;
- The type of admission;
- Comorbidities.

The cases of prolonged stays were analyzed both for the main diagnosis and for the comorbidities. The information on the diseases presented by the patient on admission and the conditions identified during the treatment was verified. This was important for the study’s reliability, as potential patients whose prolonged stays were caused by nosocomial infection were identified. For the study’s purpose, the nosocomial infection was defined as an infection related to the health services provided to the patient which occurred within 48 to 72 h following the service’s provision [21]. Based on an analysis of the occurrence of infections, a group of patients whose prolonged stays were associated with an infection experienced during the hospitalization was identified. This group constituted 6% of all the patients with prolonged stays in the department. In these cases, the hospital stays lasted far longer when compared to the other patients in the study population. On average, the duration of stay for the patient with a confirmed nosocomial infection was longer than for other patients (with a defined prolonged stay) by 22.7 days. Therefore, all these cases caused a significant overestimation of the financial components and changed the assessment of the organizational components. As a result, a decision was made to exclude the records of these patients from further stages of the study, which was intended to identify organizational wastes affecting the length of hospital stay.

### 2.5. Application of Lean Management tools

The further activities were performed using the Lean Management tools and principles. The essential principle related to creating value in the process [22], its optimization and elimination of wastes was particularly important. The study used the DMAIC (Define, Measure, Analyze, Improve, Control) methodology of process improvement and cost reduction [9]: identification of the problem (Define), its measurement and analysis (Measure, Analyze) followed by its improvement (Improve). The last stage, i.e., Control (which involves verification of the implemented solutions), was not included in the research. In addition to the analysis of the records, activities involved direct observations, mapping of specific components of the patient’s hospitalization process, the A3 reports and the Ishikawa diagram.

A key element of the study included the performance of actions in a place where value is created (Gemba Walk); therefore, observations were conducted directly at the neurology department and at the Diagnostic Imaging Unit with the staff involved in the study [23]. The A3 reports containing descriptions of current problems and their possible identified causes were developed. Next, an analysis of this documentation was performed, feasible solutions were described and potential barriers for their implementation were verified. The reports were developed according to another essential Lean Management principle, i.e., kaizen (continuous improvement). The use of the PDCA (Plan–Do–Check–Act) cycle, which consists of standardizing and optimizing processes in a controlled and continuously monitored manner, was important here [24]. The plan of action was divided into 4 basic areas:

1. Plan—involving defining the problem, its preliminary analysis, setting a feasible goal (according to the SMART principle) [25,26], searching for causes of the problem (using the 5Why tools) and initial development of the remedies.
2. Action—describing necessary measures to be taken in order to test the assumptions and solve the problem (eliminate the waste).
3. Verification—checking the validity and effectiveness of the actions taken.
4. Implementation—standardizing the positively verified solutions.

The reports were developed separately for the main process and for each analyzed subprocess. A report template is presented in Supplementary Materials.

The reports and the authors’ observations formed the basis for further analysis of the issue of prolonged stays and for verification of the cause–effect relationships.

### 2.6. Surveys among the Medical Staff

For the study’s purpose, surveys were conducted among the medical staff of the department to identify potential problems which may cause prolonged hospitalization. The survey was developed following consultations with the medical staff who, during direct observations, formulated the first issues related to organization of work that might affect the length of a patient’s hospital stay. The survey reported information on what could have an impact on prolonged stays, which enabled the classification of causes into main categories consistent with the Selker taxonomy adopted during the study and involving the stay-prolonging factors [27]:

- Diagnostic delays (imaging scans);
- Delays related to reports of imaging findings;
- Delays related to necessary additional procedures;
- Non-medical delays (no available places in care facilities, inability to transfer patients to other departments and transport) [28].

Twelve physicians and thirteen nurses and caregivers participated in the survey, constituting over three quarters (86%) and a half (54%) of the department’s employees, respectively. The survey consisted of 9 items: 7 closed-ended and 2 open-ended questions. The questions referred to possible causes of prolonged stays in the department and the average time of preparing reports of current imaging scans. In addition, the survey contained questions about retesting and reasons for it and about the stays for “social reasons”. Moreover, the respondents could present their own observations and/or suggestions which, in their judgment, might have been the causes of or the solutions to the prolonged stay problem.

**Figure 1:**
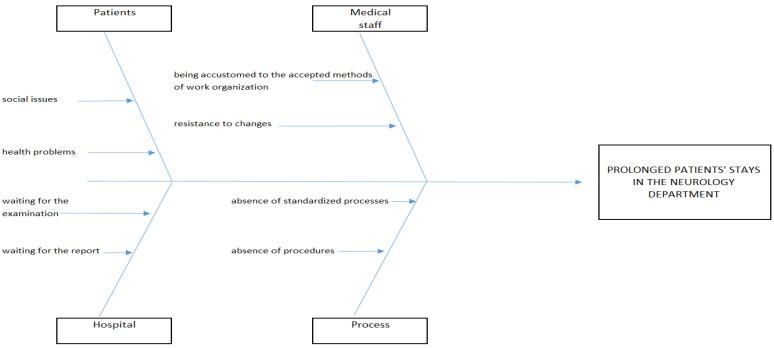
The Ishikawa diagram—analysis of causes of prolonged stays at Department III.

The next part of the research process was the verification of the identified causes of the problem with the 5Why method. Each of the causes of patients’ prolonged stays in the department was further analyzed and, by means of consecutive questions, the root cause of the specific problem was discovered. Based on the collected information, the key factors affecting the prolonged hospital stays were identified. The completed Ishikawa diagram is presented in Figure 1.

## 3. Results

### 3.1. Analysis of the Medical History Table

The medical history data of patients with over 7-day stays in the department were carefully analyzed. The analysis covered both the financial aspects (the costs of prolonged stays) and the medical data (the patient’s age, information about possible infection and comorbidities).

Based on the study’s population data (excluding nosocomial infections), the financial data were verified. They show that in 2019, the costs of prolonged stays in Department III amounted to EUR 956,498.09 (PLN 4,504,914.7) and the revenues amounted to EUR 745,355.09 (PLN 3,510,473.40), constituting 36% of the overall costs and 31% of the overall revenues, respectively. The overall financial loss for the department as a direct result of prolonged stays amounted to EUR -211,142.99 (PLN −994,441.30).

The above data clearly show that the patients’ prolonged stays generate significant costs for the department and may be a reason for financial problems of healthcare units. Therefore, it is important to find their causes and to eliminate the problems which influence prolonged hospitalization.

### 3.2. Analysis of Survey Results and Direct Observations

**Scheme 3:**
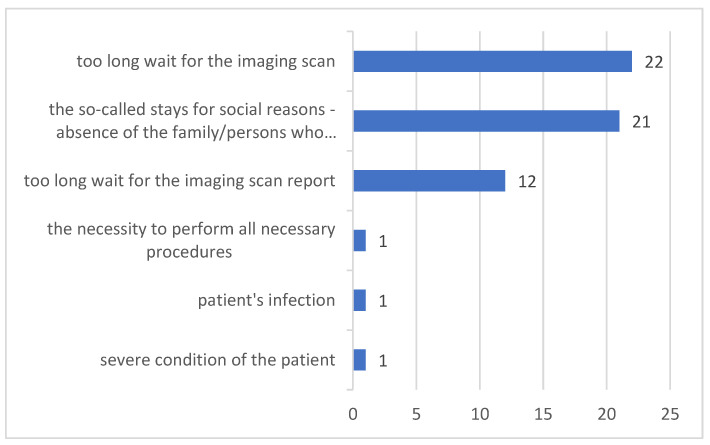
Main causes of the prolonged stays.

An extremely important part of the study was the cooperation with the health-service-providing personnel in the neurology department of the hospital. Direct observations were conducted at locations which create value in the process, i.e., in the department itself and in the Diagnostic Imaging Unit. Moreover, the neurology department staff participated in the survey. The findings show that in the respondents’ opinion, possible causes of patients’ prolonged stays in the department include excessive imaging scan waiting time (22 responses) and stays for “social reasons” (21 responses). In the respondents’ judgment, the factors affecting the length of patient’s stay, in addition to the long waiting time for a magnetic resonance imaging (MRI) scan, include nosocomial infections, inability to transfer patients to internal medicine departments for the treatment of comorbidities following the neurological diagnostic assessment, and diagnostic equipment failures. The respondents did not mention a long waiting time for a computed tomography (CT) scan or its report. The main causes of prolonged stays listed by the survey participants are presented in Scheme 3. The question could be replied to by selecting many different answers or providing own answers. Therefore, the chart presents the numbers of responses to the particular questions.

**Scheme 4:**
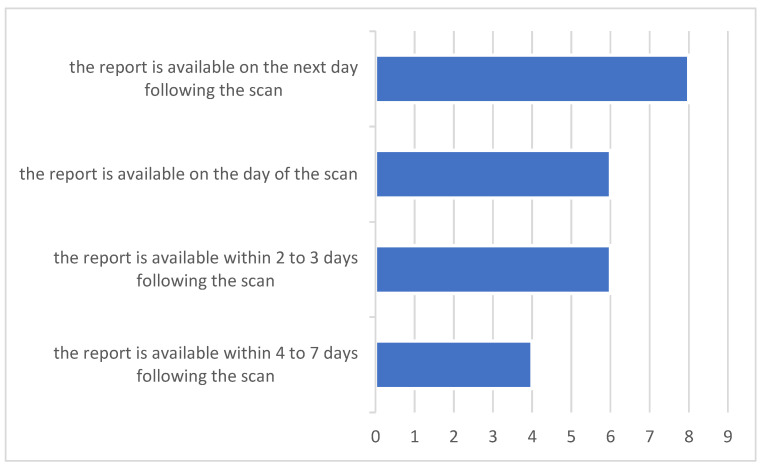
The magnetic resonance imaging scan report waiting time.

The staff participating in the survey was also asked about the imaging (both CT and MRI) scan reports’ waiting time. The responses show that the CT scans are performed without delay and the reports are delivered immediately. However, there was a problem with the MRI reports, as the responses varied and mentioned 2 days to even 7 days of necessary waiting time. The waiting time for the reports, in the opinion of the respondents, is presented in Scheme 4.

Moreover, considering responses to the next question, 9 out of 24 respondents claimed that the scan had to be repeated in many cases. The distribution of responses is presented in Table 3.

The respondents claimed that the main cause of necessary repetitions of scans was an inaccurate/unclear report of the MRI scan (14 responses). The next reasons were improperly performed scans (five responses) and unavailability of the results of previously performed diagnostic assessments (three responses). The respondents could also answer the question about the reasons for the need for repeating the scan when they marked the response other than “often” in the previous question about the frequency of necessary scan repetitions. Therefore, the total number of indicated reasons for the need for repeating the scans is higher than the number of respondents mentioning the need itself.

In the last question, the respondents could describe their own observations and suggested solutions of the prolonged stays problem. The main causes of prolonged stays mentioned by the respondents were distant scheduled dates of MRI scans, unclear reports of imaging examinations and stays for “social reasons”. They also suggested feasible solutions which would ensure the improvement of work and shorter patients’ stays, such as possible transfers of patients to rehabilitation departments following the neurological treatment and faster interventions of social workers. Following the collection of all the surveys, the responses were analyzed and, during the next part of the study, the organizational aspects of problems affecting the length of stay reported by the respondents, i.e., the processes of performing diagnostic imaging examinations and providing their results, were considered.

### 3.3. Problems Related to Diagnostic Imaging Assessments

#### 3.3.1. Problem with the Availability of Imaging Scans

**Figure 2:**
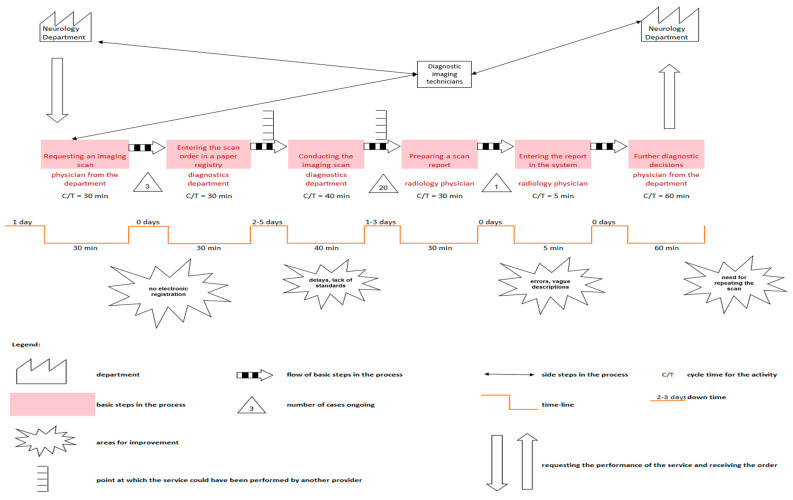
The map of the process of completing an imaging scan.

The key issue, both mentioned in the surveys and identified in the direct observations, was the unavailability of imaging examinations. To analyze this problem, a map of the process of completing an imaging scan for neurology department patients was developed. It is presented in Figure 2.

An analysis of the aspect of imaging scan completion clearly confirmed that the lengths of patients’ stays could be shortened following verification of the availability of diagnostic imaging assessment for the patients of the department. The analysis identified the bottlenecks which hampered quick diagnostic assessments:

- Lack of scheduled scan date information in the system for the ordering physician;
- The requirement to schedule the date by phone with a receptionist/technician from a specific unit (magnetic resonance imaging, computed tomography, etc.) following submission of the order in the computer system;
- Various waiting times for the MRI scan: 1 day up to even 5 days following submission of the order;
- The waiting time for the scan report: 1 to 3 days following the scan;
- Lack of simple communication regarding the cancelling/rescheduling of the scan (the receptionist phones the ordering department and leaves the information to the call recipient)—no information in the system;
- The need for rescheduling the scan date by phone after its cancellation by the employees of the Diagnostic Imaging Unit (due to, e.g., a failure of the equipment).

The analysis findings show that the computer system can be utilized more efficiently but this requires upgrading its version, adequate configuration and a training course for the users.

**Figure 3:**
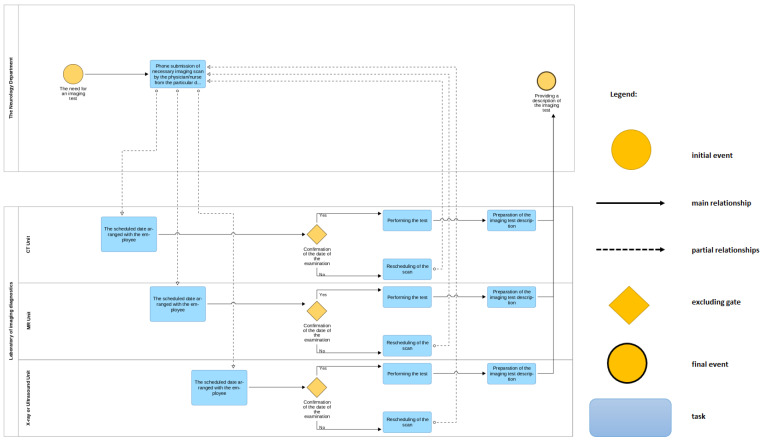
The flowchart of the imaging scan process.

The flowchart of the imaging scan process is presented in Figure 3.

**Figure 4:**
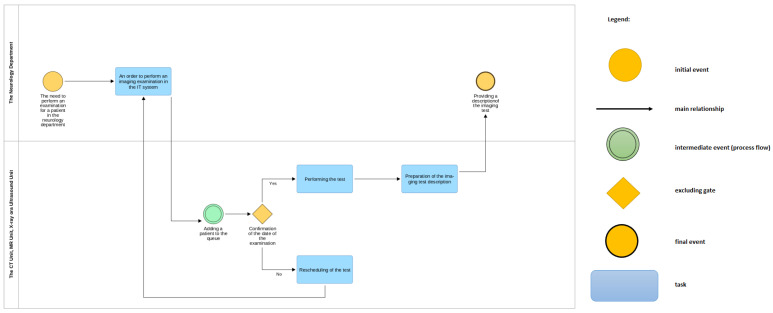
The new flowchart of the imaging scan scheduling.

A proposition of improving this aspect was put forth, aimed at shortening the imaging scan scheduling pathway. For this purpose, a team consisting of an employee of the Diagnostic Imaging Unit, an employee of the IT Unit and a representative of the investigator developed a new version of the imaging scan scheduling process and proposed the flowchart presented below in Figure 4.

This chart proposes one central site for imaging scan order submission. To avoid any errors while classifying the type of the scan and its duration, available options (e.g., contrast-dyed scans or local scans) are assigned to particular scans in the system, and based on this information, the scan duration is determined. Thus, the system blocks the scheduled hours for the ordered diagnostic assessment of a particular patient. This is a virtual system, which means that the access to the calendar view is provided both to the employees responsible for the scan’s performance and to the patients awaiting the scan.

In addition, the flowchart determines the time frame for first-priority imaging scans regarding the department where magnetic resonance imaging examinations are the most required assessments, i.e., the neurology department; here, additional hours for the patients were scheduled on Wednesdays between 3 p.m. and 7 p.m.

For the purpose of the study, a diagram of staff resources for the magnetic resonance imaging unit was also developed. In 2019, one technician was available from Monday to Friday between 8 a.m. and 8 p.m., and in addition, one nurse responsible for administration of contrast dye or anesthetics worked on Thursdays between 7 a.m. and 2.30 p.m. In the second quarter of 2022, one technician was available on working days between 8 a.m. and 4 p.m., and in addition, on two Saturdays per month (on average). The anesthetics and contrast dyes were still administered on Thursdays. During the above period, 61 and 71 MRI scans on average were performed monthly in 2019 and 2022, respectively. The number of scans for in-patients which were not performed due to a poor unit throughput could not be estimated, because of incomplete records of scans ordered in the system. In the case of external examinations, i.e., scans for individuals referred from another unit, the number of patients awaiting the scan ranged from 40 to 50 patients per month in 2022. Assuming 40 min on average for an MRI scan completion, the hospital needs another 33 effective working hours per month for an MRI technician.

Moreover, a simulation of the work schedule to ensure a more efficient unit performance with minimization of costs was performed. For the simulation purpose, equations for the estimated number of the staff necessary to secure the department per time were developed:

$$\mathrm{Np}=\left[\frac{\left(Nt1\times t1\right)+\left(Nt2\times t2\right)}{\mathrm{Ef}}\right]/7.58$$

Np—the number of stations necessary to perform a specific number of scans per 24 h.

*Nt1*—the number of the first type scans.

*Nt2*—the number of the second type scans.

*t1*—duration of the first type scan.

*t2*—duration of the second type scan.

Ef—the effectiveness factor.

7.58—the standard daily number of working hours.

$$\mathrm{Ef}=\frac{He}{Hw}$$

Ef—the effectiveness factor.

*He*—the daily number of effective working hours.

*Hw*—the overall daily number of working hours.

In addition, the following equation was used for the unit cost of the workstation provision:

$$Cp=\left[\frac{Sb+Sa+Sh+Sn}{160}\right]\times 1.1991$$

*Cp*—the cost of workstation provision.

*Sb*—the base salary.

*Sa*—the additional salary.

*Sh*—the salary for holiday hours.

*Sn*—the salary for night hours.

1991—additional insurance costs borne by the employer in Poland.

In addition, the following formula for the value of the salary for holiday hours was used:

$$Sh=\frac{Sb}{160}\times 0.45\times HHn$$

*Sh*—the salary for holiday hours.

*Sb*—the base salary.

*HHn*—the number of holiday hours.

0.45—multiplier set by the legislature for holiday hours worked out by medical staff in Poland.

Finally, the formula for the value of the salary for night hours was used:

$$Sn=\frac{Sb}{160}\times 0.65\times NHn$$

*Sn*—the salary for night hours.

*Sb*—the base salary.

*NHn*—the number of night hours.

0.65—multiplier set by the legislature for night hours worked out by medical staff in Poland.

The above equations enable the calculation of the number of employees necessary for effective performance of ordered scans and the costs of their employment. The analysis of the unit staff shows that the full utilization of the hospital resources would require more working hours for MRI technicians. However, this may generate higher personnel costs. The cost of the first 12 technicians’ working hours is EUR 93.71 (PLN 438.00). The cost of an additional 4 h will be EUR 36.37 (PLN 170.00). Moreover, performing scheduled MRI scans at night might significantly interfere with patients’ functioning in the department. Therefore, a more beneficial option is the use of the MRI unit during weekends. The cost of its availability on Saturdays and Sundays for 12 h, determined based on the above equation, will be EUR 135.85 (PLN 635.00). In addition, the estimated revenues related to 50 external MRI scans (commercial and paid by the National Health Fund) will be EUR 6,369.69 (PLN 30,000), with the additional cost of EUR 935.83 (PLN 4,407.55) regarding the estimated working hours for the technician and the physician. In the case of scans intended for in-patients, the potential profit will be the financial saving generated due to a shorter stay. Taking into account the direct and indirect costs of service provision in the department and the number of patient-days in 2019, the cost of one patient-day in the department was calculated: EUR 125.27 (PLN 590). When the optimum average time of a patient’s hospitalization in the neurology department is assumed to be 7 days, the potential savings, considering 3329 patient-days in 2019 resulting from prolonged stays in the neurology department, may reach EUR 417,023.83 (PLN 1,964,110.00). This is a general conclusion based on the research assumptions. The level of savings was calculated as the product of the number of patient-days regarding 7-day stays and the daily hospital room cost, consisting of both direct and indirect components. Shortened stays themselves will not directly generate such savings, but earlier discharges will result in more patients’ admissions and the generation of additional revenues for the facility, which will cover the costs of a patient’s stay (the daily hospital room cost); they also should generate additional profit. Moreover, a limitation of the study is the coexistence of many issues which influence the length of patient’s hospital stay. The imaging examination itself and the analysis of its results performed by a radiologist do not necessarily lead directly to an earlier hospital discharge. However, considering the need for performance of specific procedures necessary for the settlement with the National Health Fund for hospitalization of neurological patients, it is reasonable to claim that in over half of the cases, the procedures of performing the scan and preparing its report will clearly result in a shorter patient’s stay (the number of diagnoses for which several MRI scans are necessary).

#### 3.3.2. Problem with the Quality of Scan Reports

The quality of MRI scan reports was another issue mentioned by the respondents. In the hospital, the physicians preparing the scan reports are available until 3 p.m. on working days. After 3 p.m. and on non-working days, Sundays and holidays, MRI scans are sent via a teleradiology system to an external company, where the employed physicians prepare reports and send them back to the Diagnostic Imaging Unit. The hospital cannot control the choice of the medical staff that provides the services under this agreement. The only condition specified in the agreement is the need to have a medical specialization or to have started a specialization course. Direct observations and analysis of medical documentation have shown that the reports provided by teleradiology physicians were the ones requiring supplementing or correction. For the purpose of the study, all the provided reports with scan results were verified again. An analysis demonstrated that reports provided by physicians working directly with the unit constitute 82% of all performed MRI scans, while 18% of scans are sent to an external company. Therefore, a diversification of external service providers or an increase in the number of reports prepared internally was proposed.

## 4. Discussion

Prolonged stays constitute one of the key issues that affect the functioning of hospital departments [29,30], both at the organizational or qualitative level and on a financial level [31,32]. In the literature, a view is presented according to which hospitalization should be as short as possible and try to achieve the optimum use of the resources with a high quality of provided services. Excessively long hospital stays have a negative impact both on the costs of health services and on patients’ access to medical services [31]. This has been confirmed by the data from the examined hospital, where the percentage of bed occupancy in the neurology department has been 100.2% over the years (assuming standard operation of the department and patient flow, the optimum occupancy rate ranges from 80% to 85%). This means that the number of patients requiring immediate neurological intervention is so high that they are admitted despite full occupancy, or that it is necessary to refer them to another center when hospitalization in the department is not possible due to organizational issues. More effective treatment in the neurology department will result in greater availability of beds and treatment options.

The literature analysis shows that the length of a patient’s stay in a department greatly depends both on the efficiency of the hospitalization process and on the standardization of clinical pathways [33]. Thus, it is very important to develop procedures to simplify basic activities performed by the medical staff and to help avoid waste of time. Initiatives of various organizations demonstrate that these are very important aspects of the provision of health services [34]. Moreover, research shows that it is possible to increase the number of patient admissions based on the organizational capacity of an institution. However, ongoing monitoring and improving patient flows, understanding the operation of the system and focusing on activities performed directly during patients’ hospitalization and on additional processes whose functioning ensures shorter hospital stays (rehabilitation, transport and out-patient care) are necessary [2]. Moreover, the implementation of a new method of hospital bed management, where the standardized reports and the activities of specific organizational units enable the central management of bed availability and quick interventions in the case of emerging problems, may contribute to better efficiency and performance of healthcare institutions [2].

**Scheme 5:**
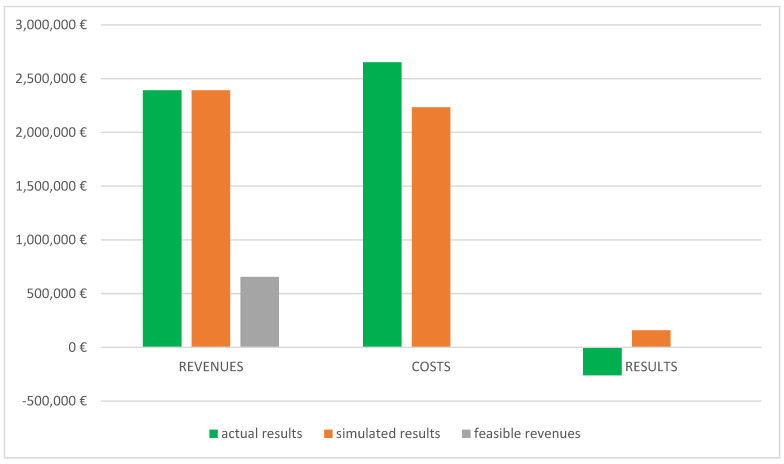
The financial loss for the department and the simulation of financial result.

The LM tools applied in the management of healthcare sector units enable the identification of waste and feasible solutions [18]. The methods used in the research process led to the identification of a problem which generated high losses for the hospital, i.e., prolonged patients’ stays in the department. Nevertheless, a simulation of department operation after implementation of the organizational solutions intended to achieve shorter stays lets us assume estimated lower costs and therefore improved financial results. The shortening of hospitalization time enables admitting more patients and thus financing of fixed costs, which are borne by the hospital regardless of the LOS. The costs of hospital rooms and of physician and nursing care amount to EUR 125.47/person-day. In the studied period, the total number of hospitalization stays exceeding 7 days in the department amounted to 3329. Assuming the admission of new patients, the costs could be reduced by EUR 417,696.43 annually. The financial loss associated with the identified waste and the estimated simulation of the financial results with savings related to shorter stays are presented in Scheme 5.

The methods of loss elimination presented in further research stages referred to organizational activities intended to improve particular processes in the hospital. In addition, activities directly related to the patient and to the socioeconomic factors, such as development of treatment strategies, community interviews or patient referrals to other services/centers, are possible [35].

The activities of the hospital aimed at improving the quality of patients’ care and shortening their hospital stays focus on determination of the pathway of patients’ care through care coordination, creation of multidisciplinary teams and optimization of resources’ use [36]. While planning a patient’s clinical pathway, it is very important to identify and eliminate the “bottlenecks” and to improve the value flow in the process [36]. In the literature, a view is presented according to which it is possible to shorten a patient’s stay and to reduce the treatment costs by implementing standardized pathways adjusted to the needs and potentials of a specific organization [37,38,39].

Many studies confirm that the use of Lean Management tools in the management of a healthcare unit leads to improvements in the quality of services by improving processes and eliminating waste [40,41,42,43]. The analyses demonstrate that the implementation of changes in work organization might contribute to shorter patients’ hospital stays and to reduced costs of hospitalization. However, many literature reports emphasize that in the case of implementation of modern tools, hospital managers may encounter employees’ reluctance and resistance to changes [44,45]. The conducted study showed that the tasks associated with imaging scans were performed according to the pathways established by a single employee. Therefore, the process of scan scheduling was a complex, multistage activity. The developed new system will require an active involvement of both the IT employees and the Diagnostic Imaging Unit staff.

The other issues affecting the success of LM tool implementation are the complexity of hospital processes [46] and the specifics of the healthcare sector and institutions (saving human health and life, a complex organizational structure and diverse ranges of activities performed by specific units) [42]. While analyzing the studied department, the authors focused on issues which are potentially common to many hospitals, regardless of their type or location. A prolonged patient stay generates unnecessary costs, as it poses the risk of nosocomial infections which must be treated. It also has an impact on social costs such as the unavailability of beds for the next patients who require medical care. In many cases, the problems associated with prolonged hospitalization are organizational issues which can be solved by an analysis according to the scheme proposed in this study. The equations intended for calculations of costs of department’s staff provision were developed in accordance with Polish regulations. However, they represent a universal method of thinking about personnel-related costs and a way for analyzing them.

A limitation of the study was the inability to evaluate the presented solutions intended to improve the process of performing and reporting the results of diagnostic assessments. They may constitute an auxiliary component and indicate a method of formulating conclusions and of preparing improvements in an organization.

Moreover, the analysis of the sources of the issue of repeated scans and their reports, which was based on subjective opinions provided by the respondents, was a qualitative assumption and did not have a quantitative character. In order to obtain statistical data, it would be necessary to use a control sheet and to systematically collect data over a longer period.

## 5. Conclusions

This study enabled the identification of factors affecting the length of a patient’s stay in the hospital. Moreover, the analysis showed that prolonged hospital stays resulted in higher costs for the institution.

In the literature, a view is presented according to which in the process of provision of services, a patient’s admission and discharge should coincide with the beginning and the end of the clinical process [47]. In this situation, hospitalization is efficient both in the medical context and in the organizational and financial aspects. Additionally, considering high costs of healthcare services, shortened patients’ stays should be reflected in hospitals’ financial results due to cost reductions and a higher diagnostic potential for the next patients.

The study findings identify potential bottlenecks of a patient’s hospitalization process and show the methods which can help conduct a similar research process in another institution. The next stages present the applied tools and the method of formulating conclusions based on the findings. These are universal methods of identification, collection and analysis of data which can be applied in various units of the healthcare sector. The application of Lean Management tools in the study enables us to verify the main problems leading to prolonged hospital stays and to develop feasible solutions. The financial analysis showed that slight changes of organizational issues might contribute to improvement of the unit’s financial situation. In addition, as the study demonstrated, the generation of profit requires in some cases additional costs which will ultimately provide higher revenues.

An intermediate effect of the elimination of factors which impact prolonged stays of the patients was an improvement of medical and logistical efficiency (shortening the time needed for examination, elimination of repeated examinations and therefore decreasing patients’ exposure to the harmful impact of diagnostic imaging devices, shortening the treatment procedure, faster return of the patients to their homes and to physical activity, and lower exposure of the patients to the risk of infection). Shortening patients’ stay at a department with an occupancy rate of 100% allows the admitting of the next patients and shortening the bed waiting time, which is a particularly important aspect for neurological patients.

The above conclusions confirm the thesis that the application of Lean Management tools may contribute to improvement in the functioning of hospitals and other institutions of the healthcare sector [40]. Further studies on the application of Lean Management in the healthcare area should focus on the development of a method to evaluate the efficiency of the implemented solutions intended to shorten a patient’s hospital stay.

## Figures and Tables

**Table 1:** Process of conducting the study.

No.	Stage	Actions/Results
1.	Defining the concept of prolonged stay	Analysis of literature and Central Statistical Office data as a result of which it was assumed that a prolonged hospitalization was one that lasted over 7 days.
2.	Analysis of data concerning the length of stay at individual departments with an analysis of their financial results Identification of the department at which the study will be conducted and the period from which documentation will be tested	Selection of the department for study—Neurology Department III—in accordance with the assumed criteria: (1) average monthly occupancy rate was 100%, (2) high financial loss (at a level of EUR 259,323.33), (3) high share of prolonged stays (44% of the total). Data from the years 2020 and 2021 were not included in the analysis due to the hospital’s transformation intended to provide beds for COVID-19 patients.
3.	An analysis of the most frequently occurring diagnoses groups in order to establish the scope of documentation for further study	Analysis of medical documentation of patients from the period of January–December 2019 (1795 items). Exclusion criteria included stays related to the performance of drug programs and one-day stays (diagnostic stays). Documentation of 1116 patients was selected for the next stage. Data were divided by groups of illnesses (in accordance with ICD-10). Groups constituting 80% of all diagnoses were selected for the study (888 items). Then, cases where hospitalization exceeded 7 days were selected (624 items), and using an ABC table 5 groups of diagnoses with the largest share of prolonged stay cases were selected (a total of 504 cases).
4.	Analysis of the patients’ medical documentation and exclusions	Sorting the medical documentation taking into account patient data (age, sex and comorbidities), financial data (revenue and hospitalization cost) and logistical data (type of admission). An adverse event exclusion criterion was accepted. Nosocomial infections which resulted in a significant prolongation of the stay and thus could hinder the verification of the occurrence of other reasons for prolonged hospitalizations were identified. During the study, 29 cases were identified, the documentation of which was excluded from the next stage of analysis.
5.	An analysis of the prepared documentation in order to verify the causes for extended hospitalizations at the department selected for the study	Areas were identified related directly to patient’s situation (e.g., polymorbidity) and to the need to provide care (“social stays”). Verification of the financial data has demonstrated that the share of the financial result generated by patients with prolonged stay constitutes over 80% of the general financial result of the department at the period under analysis.
6.	Identification of wastes and of reasons for prolonged stays and their analysis	LM tools were used to identify wastes occurring in the process of hospitalization of a patient and possible reasons for prolonged stays (VSM, Ishikawa Diagram, A3 Report). Surveys were conducted with the medical personnel. The analysis of the obtained data enabled us to define the main problems and to indicate the bottlenecks which occur during the hospitalization process.
7.	Propositions of possible solutions with analysis of potential benefits	Proposals of solutions which were possible to implement were prepared (e.g., a change in the process of registering the patients for scans—a central site in the IT system, diversification of services of physicians preparing imaging scan reports). A simulation of the financial result which could be improved by shortening the stay duration as a result of reduction in costs and increase in revenue was also conducted.

**Table 2:** A list of study-specific diagnoses.

No	Diagnosis	Number of Cases	Number of Cases, Incrementally	Percentage	Lorenz Curve
1	I63 Cerebral infarction	269	269	24%	24%
2	G40 Epilepsy	141	410	13%	37%
3	G45 Transient ischemic cerebral attack and related syndromes	128	538	11%	48%
4	G35 Multiple sclerosis	62	600	6%	54%
5	I61 Intracerebral hemorrhage	40	640	4%	57%
6	I69 Sequelae of cerebrovascular disease	34	674	3%	60%
7	G37 Other demyelinating diseases of the central nervous system	34	708	3%	63%
8	G62 Other polyneuropathies	34	742	3%	66%
9	D43 Neoplasm of uncertain or unknown behavior of brain and central nervous system	33	775	3%	69%
10	M48 Other spondylopathies	26	801	2%	72%
11	R56 Convulsions, not elsewhere classified	24	825	2%	74%
12	C79 Secondary malignant neoplasm of other and unspecified sites	18	843	2%	76%
13	C71 Malignant neoplasm of brain	15	858	1%	77%
14	I67 Other cerebrovascular diseases	15	873	1%	78%
15	C93 Polyneuropathy in diseases classified elsewhere	15	888	1%	80%

**Table 3:** The causes of necessary repetitions of magnetic resonance imaging scans.

	Number of Responses
The need for repeating the scan	9
Reasons of repeated scans:	
- an inaccurate/unclear report of the scan	14
- the scan was performed improperly	5
- the previous diagnostic assessment results are unavailable	3

## Data Availability

The data presented in this study are available on request from the corresponding author.

## Supplementary Materials

The following supporting information can be downloaded at: https://www.mdpi.com/article/10.3390/ijerph20021067/s1, A3 Report.
