# Supplementary material for: Implementation of Lean Management Tools Using an Example of Analysis of Prolonged Stays of Patients in a Multi-Specialist Hospital in Poland
Source: Int J Environ Res Public Health. 2023 Jan 7;20(2):1067. doi: 10.3390/ijerph20021067 (PMC9858728; doi:10.3390/ijerph20021067)
Supplement: Supplementary file 1 [file ijerph-20-01067-s001.zip › ijerph-2112111-supplementary.pdf]

DEPARTMENT:<br>PROCES:	REPORT A3	DATE: LEADER:<br>PARTICIPANTS:

1) DEFINITION OF THE PROBLEM	5) DEVELOPMENT OF REMEDIAL MEASURES
2) ANALYSIS OF THE PROBLEM	6) IMPLEMENTATION<br>**DO**
3. SETTING A TARGET<br>**PLAN**	7) VERIFICATION<br>**CHECK**
4) SEARCHING FOR THE CAUSES OF THE PROBLEM	8) STANDARDISATION<br>**ACT**
